# Fungal endophyte infection of ryegrass reprograms host metabolism and alters development

**DOI:** 10.1111/nph.13614

**Published:** 2015-08-25

**Authors:** Pierre‐Yves Dupont, Carla J. Eaton, Jason J. Wargent, Susanne Fechtner, Peter Solomon, Jan Schmid, Robert C. Day, Barry Scott, Murray P. Cox

**Affiliations:** ^1^ Institute of Fundamental SciencesMassey UniversityPalmerston North4442New Zealand; ^2^ The Bio‐Protection Research CentreMassey UniversityPalmerston North4442New Zealand; ^3^ Institute of Agriculture and EnvironmentMassey UniversityPalmerston North4442New Zealand; ^4^ Research School of BiologyCollege of Medicine, Biology and EnvironmentAustralian National UniversityCanberraACT0200Australia; ^5^ School of Medical SciencesUniversity of OtagoDunedin9054New Zealand

**Keywords:** endophyte, metabolism, mutualism, RNAseq, ryegrass, symbiosis

## Abstract

- Beneficial associations between plants and microbes play an important role in both natural and agricultural ecosystems. For example, associations between fungi of the genus *Epichloë*, and cool‐season grasses are known for their ability to increase resistance to insect pests, fungal pathogens and drought. However, little is known about the molecular changes induced by endophyte infection.
- To study the impact of endophyte infection, we compared the expression profiles, based on RNA sequencing, of perennial ryegrass infected with *Epichloë festucae* with noninfected plants.
- We show that infection causes dramatic changes in the expression of over one third of host genes. This is in stark contrast to mycorrhizal associations, where substantially fewer changes in host gene expression are observed, and is more similar to pathogenic interactions. We reveal that endophyte infection triggers reprogramming of host metabolism, favouring secondary metabolism at a cost to primary metabolism. Infection also induces changes in host development, particularly trichome formation and cell wall biogenesis.
- Importantly, this work sheds light on the mechanisms underlying enhanced resistance to drought and super‐infection by fungal pathogens provided by fungal endophyte infection. Finally, our study reveals that not all beneficial plant–microbe associations behave the same in terms of their effects on the host.

## Introduction

Interactions between plants and beneficial microbes are crucial to the establishment and maintenance of stable ecosystems, particularly in the face of environmental stresses. An ideal model system for studying beneficial plant–fungal interactions is the association between fungi of the genus *Epichloë* and cool‐season grasses (Schardl *et al*., 2013). Endophyte infection of grasses can increase host resistance to insect pests through protection from herbivory (Gallagher *et al*., 1984; Rowan *et al*., 1986, 1990), enhance drought tolerance (Arachevaleta *et al*., 1989; West *et al*., 1993) and give protection from super‐infection by fungal pathogens (Tian *et al*., 2008; Pańka *et al*., 2013b). Consequently, these associations have been widely exploited by the agricultural industry (Johnson *et al*., 2013; Young *et al*., 2013), and have been touted as a ‘perfect partnership’ (Christensen & Voisey, 2007). However, although much is known about the enhanced stress‐tolerance provided by fungal infection, little is known about what other effects endophyte infection may have on the host.

A small number of microarray and RNAseq analyses are starting to shed light on the effects of plant–fungal symbiotic associations on the host transcriptome. For example, in both arbuscular mycorrhizal associations (Güimil *et al*., 2005; Guether *et al*., 2009; Zouari *et al*., 2014), and ectomycorrhizal associations (Plett *et al*., 2015), infection tends to change expression of a very small subset of host genes (*c*. 1–3%). Similarly, in endophytic associations between *Trichoderma* and grapevine (Perazzolli *et al*., 2012) or *Arabidopsis* (Morán‐Diez *et al*., 2012), only *c*. 1% of host genes are altered. By contrast, the effects of pathogen infection tend to be more dramatic, with significantly more host genes differentially expressed (*c*. 20%) (Doehlemann *et al*., 2008; Kawahara *et al*., 2012; De Cremer *et al*., 2013).

In order to fully understand the impact of *Epichloë* infection on perennial ryegrass gene expression, we performed deep mRNA sequencing (RNAseq) on endophyte‐infected and endophyte‐free plants. This study furthers an earlier SOLiD‐SAGE analysis of *E. festucae* in association with red fescue grass performed by Ambrose & Belanger (2012) that identified *c*. 200 host genes which are differentially expressed during a mutualistic association. We uncover major effects on host metabolism and development, and reveal that unlike other beneficial plant–fungal interactions, which affect expression of only a small number of host genes, *Epichloë festucae* infection, under controlled growth conditions, leads to differential expression of more than a third of host genes, with changes reflective of metabolic reprogramming.

## Materials and Methods

### Fungal strains and growth conditions

*Epichloë festucae* strain Fl1 and *Lolium perenne* L. cv Samson were used in analyses presented in this study. For isolation of total RNA *in planta*, endophyte‐free perennial ryegrass seeds were germinated on 3% water agar plates and inoculated as previously described (Latch & Christensen, 1985). Plants were grown in temperature‐ and light‐controlled conditions (19**°**C, 16 h : 8 h, light : dark; Gro‐lux bulbs, Sylvania, Danvers, MA, USA.), and screened for infection as previously described (Tanaka *et al*., 2005). Plants used for metabolite, photosynthesis, chemical and microscopy analyses were independent of those used for RNA sequencing but were grown under the same controlled conditions.

### Generation of a ryegrass expressed sequence library

RNA profiles were generated for pooled tissues from both infected and uninfected plants in order to observe as many different RNAs as possible. Previous analyses have shown *E. festucae* Fl1 represents *c*. 1–2% of total biomass in infected *L. perenne* plants (Young *et al*., 2005). RNA samples were prepared for sequencing with the Illumina Inc. (San Diego, CA, USA) TruSeq^™^ RNA Sample Preparation v2 kit. Completed libraries were loaded across three lanes of an Illumina HiSeq 2000 at a concentration of 6 pM. Sequencing was done using a 2 × 100 bp protocol and the resulting FastQ files were assessed for sequence quality and adapter contamination using FASTQC v.0.10.1 (Andrews, 2010). The RNA samples used to generate the expressed sequence library are independent from those used later for transcriptomics analysis.

418 752 794 high quality reads (> Q20) were used for *de novo* assembly using Trinity v. r2012‐06‐08 (Grabherr *et al*., 2011). The *de novo* assembly generated 300 938 predicted ESTs ranging in length from 201 to 17 375 bp. 118 260 ESTs were > 1000 bp in length.

This EST collection was refined into open reading frame (ORF) predictions using the TransDecoder module (included in the Trinity package) to condense the original ORF predictions into a representative list and further refined using CD_Hit (95% identity cut‐off) (Fu *et al*., 2012) culminating in a collection of 58 303 ORFs. These were further refined by BLAST against *E. festucae* gene models (removing 8109 ORFs) and the GenBank nr database (E‐value cut‐off 1 × 10^−5^) to restrict ORFs to only those of plant origin, resulting in a final set of 42 083 host derived ORFs. A final step of manual validation was performed on all ORFs showing differential expression between endophyte‐infected and endophyte‐free plants to confirm their plant origin.

All ORFs have been deposited in the NCBI TSA database (primary accession identifier GDCY00000000, with individual ORF accession numbers GDCY01000001–GDCY01050169). The reads used to build the ORFs are available in the NCBI Sequence Read Archive (accession numbers: SRR1909335, SRR1909336 and SRR1909338).

### Transcriptomics analysis

Pseudostem tissue from plants infected with wild‐type *E. festucae*, and plants mock‐inoculated with a potato dextrose (PD) agar block as a control for wounding (referred to as uninfected) were harvested and snap frozen in liquid nitrogen. To allow direct comparison with published *E. festucae* symbiotic mutant transcriptomics analyses (Eaton *et al*., 2010, 2015), RNA was extracted from plants 7 wk post‐inoculation. Total RNA was isolated from 0.5 to 0.6 g (fresh weight) tissue from a minimum of two plants for each of the two biological replicates using TRIzol reagent^®^ (Invitrogen). mRNA libraries were generated by Cofactor Genomics, and a 100‐nucleotide single‐end run was performed according to Illumina guidelines. Libraries were indexed, then pooled and sequenced on an Illumina HiSeq 2000, and the raw results were processed using Casava v.1.8.2 (Illumina Inc.). Read quality was checked using the SolexaQA package (Cox *et al*., 2010). Using the same package, reads were trimmed such that all bases had a probability of error ≤ 0.05, and only reads ≥ 80 bases long were retained. Reads were mapped for each biological replicate separately to the set of *L. perenne* ORFs described above (*n* = 42 083) using the Burrows‐Wheeler mapping algorithm implemented in Bowtie2 v.2.0.2 (Langmead *et al*., 2009). Reads that did not map uniquely to a single reference were excluded, and the number of reads that mapped to each ORF was determined. Statistical significance, accounting for the variance between replicates, was calculated using Fisher's Exact Test as implemented in the R package DEGseq v.1.13.3 (Wang *et al*., 2010). A correction for multiple testing was applied using the False Discovery Rate (FDR) approach (Storey & Tibshirani, 2003). The fold difference for each gene *i* was calculated from the raw read counts normalised by the total number of mapped reads as

(Eqn 1) $$FD_{i}=\frac{\max(inf_{i},uninf_{i})}{\min(inf_{i},uninf_{i})}$$

In the endophyte‐infected sample, plant reads accounted for 96.5% of the total reads, so the effect of fungal reads on host gene coverage was considered negligible and no further normalisation was performed to account for this mixed transcriptome. FASTQ files generated for endophyte‐infected and endophyte‐free samples have been submitted to the NCBI Sequence Read Archive (accession numbers SRR1585306–SRR1585309).

The RNAseq results were confirmed using the Nanostring nCounter GX protocol (Nanostring Technologies Inc., Seattle, WA, USA). Using the same RNA used for RNAseq analysis, 100 ng total RNA in 5 μl total volume was processed using the standard nCounter total RNA protocol. The Nanostring CodeSet (reporter and capture probes that hybridise to the target sequences of interest, forming a tripartite complex) was designed as 100 nt sequences unique to each gene. The uniqueness of each probe was determined using FASTA v.36.3.6 (Charlottesville, VA, USA) with a Smith‐Waterman search of the probe on all targets in the genome. The genes of the CodeSet were chosen to cover a large range of expression and fold values. Genes where a unique probe sequence was hard to find were avoided. The total RNA and CodeSet were combined with hybridisation buffer and incubated at 65**°**C for 20 h. Hybridised samples were processed in batches of 12 using the robotic prep station (high sensitivity protocol, 3 h per 12‐sample cartridge). Data acquisition was performed using the GEN2 digital analyzer, with the ‘max’ field of view setting (555 images per sample; 5 h scan per cartridge). Raw count data were exported as RCC files from the digital analyzer, and quality control checked by using Nanostring's nSolver data analysis tool (default QC settings). Five housekeeping genes were included in the CodeSet. The housekeeping genes were determined as genes displaying minimal changes (ratio ≈ 1) in different RNAseq analyses performed on the system described in this paper, including four different mutants of the *Epichloë* endophyte. To normalise the gene expression, the count for each gene was divided by the median of the counts of the housekeeping genes. The same operation was performed on the RNAseq results. The infected vs uninfected ratios were compared using a Spearman's rank‐order correlation.

### Functional annotation

Functional annotation of the gene set was performed using Mercator (Lohse *et al*., 2014), Pfam (Punta *et al*., 2012), Blast2GO (Conesa *et al*., 2005) and BLAST (Altschul *et al*., 1990; Zhang *et al*., 2000) (Supporting Information Table S1). All genes involved in the pathways/processes presented in this study were manually verified to confirm functional prediction and classification. As the library contains sequences from many sources (plant, fungi, bacteria, etc.), a BLAST search was performed to determine the likely origin of each sequence. The nucleotide (nucleic acids) and nonredundant (proteins) databases of NCBI (versions downloaded on 23 January 2013) were used to screen for a wide range of potential origins, with an E‐value threshold of 1 × 10^−5^. All annotations were verified manually and used to infer the taxonomic origin of the sequences. To associate Pfam domains to sequences, InterProScan5 (Quevillon *et al*., 2005) and a Perl script pfam_scan.pl freely available on the Pfam database (Punta *et al*., 2012) were used. GO terms for each coding sequences were determined using InterProScan5 and Blast2GO (Conesa *et al*., 2005). GO term enrichment analysis was performed using Blast2GO combined with a two‐sided Fisher's exact test with false discovery rate correction. All sequences were analysed using the MapMan classification tool (Thimm *et al*., 2004; Usadel *et al*., 2005, 2009; Urbanczyk‐Wochniak *et al*., 2006) of Mercator (Lohse *et al*., 2014), which automatically computes functional information from sequence homology. MapMan allows large datasets to be mapped onto simple schemes representing pathways or other processes. Unfortunately, these schemes were often insufficiently precise for our system and induced interpretation errors due to the repetition of some genes. To overcome this problem, the functional classification information provided by Mercator was manually screened to remove any misclassifications and duplicates, and then manually organised into pathway or process groupings.

### Metabolomics analysis

Apoplastic fluid was isolated from endophyte‐infected and endophyte‐free perennial ryegrass plants 8 wk post‐inoculation using a modification of the method of van Hove *et al*. (2002). Cut pseudostem and blade tissue was immersed in sterile distilled water in a 50‐ml syringe and vacuum infiltrated by repeated cycles of 30 s pulling on the plunger, followed by 30 s pushing on the plunger. This was repeated until the tissue became dark green and sunk inside the syringe during pressure application. The tissue was then surface dried and placed inside a 1‐ml pipette tip. Filled tips were then placed inside a 50‐ml falcon tube and centrifuged at 2195 ***g*** at 4°C for 20 min. The apoplastic fluid was collected from the bottom of the tube and stored at −80°C.

Primary metabolite analysis was performed on the apoplastic fluid samples as previously described (Vincent *et al*., 2012). Briefly, freeze‐dried apoplastic fluid samples were derivatised inline (Halket & Zaikin, 2006) with the Gerstel MultiPurpose Sampler. Methoximation of carbonyl groups was achieved with incubation and shaking at 37°C (90 min, 1000 ***g***) with methylamine‐HCl (20 μl, 20 mg ml^−1^ in pyridine). Trimethylsilyation of polar groups was completed by addition of 30 μl of *N*‐trimethylsilyl‐*N*‐methyl trifluoroacetamide (MSTFA) following by incubation at 37°C and shaking for 30 min. GC‐MS analysis was then performed as previously described (Lowe *et al*., 2009). Samples were analysed in splitless and 20 : 1 split modes in order to obtain quantitative data for all metabolites over the extensive range of concentrations present. The splitless data set was used for metabolites of lower concentration, which were not reliably quantified in the split analysis. Metabolite abundances were normalised to the ribitol internal standard and sample weights using AnalyzerPro (SpectralWorks Ltd, Runcorn, UK). Mass spectra of compounds found were identified using the publicly available Golm metabolome database (Max Planck Institute for Plant Physiology) (Schauer *et al*., 2005) and the commercial National Institute of Standards and Technology 08 (NIST, http://www.nist.gov/index.html) mass spectral library. Data analyses were carried out using the JMP v8.0.1 statistical package (SAS Institute Inc., Cary, NC, USA).

### Photosynthesis and stomatal conductance analysis

Light‐saturated net exchange rate of CO_2_ (light‐saturated net photosynthetic rate or *P*_max_) and stomatal conductance were characterised in plants at 14 wk post‐inoculation (*n* = 6 plants each for endophyte infected and uninfected). Measurements were carried out with an LI‐6400 infra‐red gas analyser (Li‐Cor Inc., Lincoln, NE, USA), where cuvette conditions were maintained at a saturating 500 μmol m^−2^ s^−1^ PAR, 75% relative humidity, 20°C cuvette block temperature and 400 ppm of CO_2_. During measurements, groups of blades from each plant were placed parallel to each other, but not overlapping, in order to fulfil the leaf area requirement of the cuvette. Tillers were maintained in the cuvette for at least 15 min to reach steady‐state before measurement. Following measurement, cuvette‐exposed tiller regions were dried to a constant mass in order to express net photosynthesis per unit leaf dry weight (DW), and therefore normalised for any variation in leaf thickness between tillers.

### Phenylpropanoid and chlorophyll extraction

In order to examine differences in phenylpropanoid levels between infected and uninfected plants, sections of pseudostem were taken from the base of tillers 9 wk post‐inoculation and snap frozen in liquid nitrogen (*n* = 4 plants each for endophyte infected and uninfected). Approximately 0.2 g of tissue was ground in liquid nitrogen, and *c*. 0.1 g transferred to an eppendorf tube (exact mass recorded). The tissue was resuspended in 1 ml of acidified methanol (79 : 20 : 1; methanol : water : HCl) and mixed for 30 min in the dark. Cellular debris was pelleted by centrifugation at 16 200 ***g*** for 15 min. Absorbance was measured at 524 nm for anthocyanins (undiluted extract) and 300 nm for flavonoids (10 fold diluted extract). The absorbance was then normalised based on the fresh weight.

In order to examine differences in the levels of chlorophyll between infected and uninfected plants, sections of blade tissue were taken 9 wk post‐inoculation and snap frozen in liquid nitrogen (*n* = 3 plants each for endophyte infected and uninfected). Approximately 0.2 g of tissue (exact mass recorded) was ground in 10 ml ice‐cold methanol. The suspension was then centrifuged at 2404 ***g*** at 4**°**C for 5 min. The supernatant was transferred to a fresh tube and the pellet re‐extracted twice with 5 ml ice cold methanol. The absorbance was measured at 652, 663 and 646 nm (five‐fold diluted extract). Absorbances were normalised based on the fresh weight. Absorbance at 710 nm was measured to confirm the absence of compounds, which interfere with the measurement of chlorophyll.

### Microscopy

For examination of chitin localisation, leaf sheath tissue from close to the base of wild‐type *E. festucae* infected tillers (20 wk post‐inoculation) was cleared overnight in 95% ethanol at 4**°**C then permeabilised by incubation in 10% potassium hydroxide overnight at 4**°**C. Tissue was vacuum infiltrated with 1% aniline blue, 10 μg ml^−1^ WGA‐AF488 Alexafluor (Molecular Probes, Eugene, OR, USA) for 1 h, and observed using a Leica SP5 DM6000B confocal microscope (Leica Microsystems GmbH, Wetzlar, Germany).

For examination of plant cell wall structure, *c*. 0.5 mm‐thick sections of pseudostem tissue (11 wk post‐inoculation) were prepared as previously described (Eaton *et al*., 2010). A minimum of 60 cell walls, from two independent plants, were measured for endophyte‐infected and uninfected samples. Samples were examined using a FEI Tecnai G2 Spirit BioTWIN electron microscope. Cell wall thicknesses were measured using ImageJ (Schneider *et al*., 2012).

For examination of trichomes using scanning electron microscopy, leaf blade samples were taken 2 cm from the leaf tip (21 wk post‐inoculation) and prepared as previously described (Eaton *et al*., 2010). A minimum of 11 trichomes, from two independent plants, were measured for endophyte‐infected and uninfected samples. Samples were observed using an FEI Quanta 200 scanning electron microscope. Trichome area was measured using ImageJ.

## Results

### Identifying endophyte‐induced changes in perennial ryegrass gene expression

**Figure 1:**
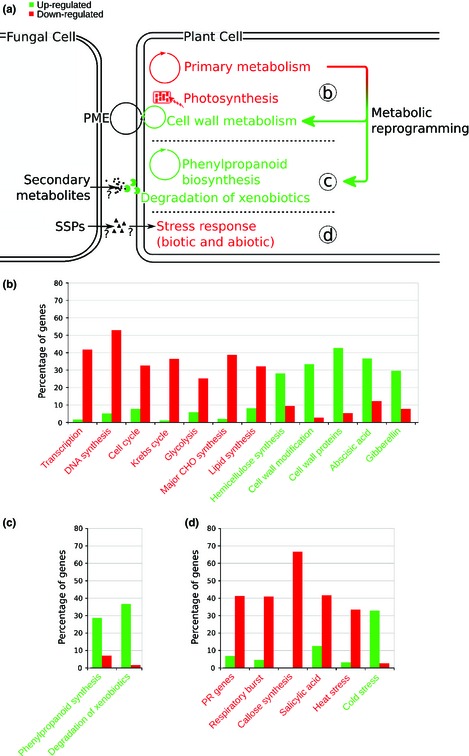
Summary of endophyte‐induced changes in host gene expression. (a) Schematic summarising the major changes in gene expression induced by endophyte infection. Red text indicates those processes largely downregulated in infected plants, green text indicates those processes largely upregulated in infected plants. PME, pectin methylesterase; SSPs, small secreted proteins. Colours apply to all sections. (b) Graph summarising changes in key sets of genes involved in primary metabolism. CHO, carbohydrate. (c) Graph summarising changes in key sets of genes involved in secondary metabolism. (d) Graph summarising changes in genes involved in host responses to biotic and abiotic stresses. PR, pathogenicity related.

In order to identify perennial ryegrass genes whose expression is altered in response to infection by *E. festucae*, high‐throughput mRNA sequencing was performed on two biological replicates each for wild‐type *E. festucae*‐infected plants and uninfected control plants. Ryegrass gene sequences were first generated by *de novo* assembly of RNAseq reads, followed by extraction of the longest coding sequence. This resulted in identification of 42 083 unique sequences. Given that *E. festucae* infection is generally asymptomatic in the absence of biotic or abiotic stresses, and as mycorrhizal associations have limited impact on their host transcriptome (Güimil *et al*., 2005; Gomez *et al*., 2009; Guether *et al*., 2009; Zouari *et al*., 2014), few effects of endophyte infection were expected in the host grass transcriptome (Table 1). Surprisingly, 38% of the predicted ryegrass gene set (*n* = 16 041) was found to be differentially expressed, with statistically significant differences and a two‐fold expression change cut‐off. The majority of these genes (66%; *n* = 10 532; 25% of all differentially expressed genes) were downregulated in infected plants. Using the Mercator pipeline (Lohse *et al*., 2014) combined with manual annotation (verification of sequence homology, predicted function and pathway classification), functional predictions were made for *c*. 50% (*n* = 8061) of these genes. This revealed changes in three key areas detailed below: primary metabolism, secondary metabolism and stress‐related gene expression (Fig. 1; Table S2). The RNAseq results were verified using a Nanostring nCounter GX analysis on a subset of the host genes covering a large range of expression and fold ratios. The Spearman's rank‐order correlation is *r*_s_ = 0.90, showing that the observed results are not an artefact of the RNAseq method (Table S3).

**Table 1:** Comparison of the transcriptomic effects of different beneficial and pathogenic fungi on their hosts

Beneficial/pathogen	Host	Fungus	Association type	Trend	DEG^a^	Analysis	Reference
Beneficial	Perennial ryegrass	*Epichloë festucae*	Endophyte	Down	38%	RNAseq	This study
Beneficial	Rice	*Glomus intraradices*	Mycorrhiza	Up	0.5%	Microarray	Güimil *et al*. (2005)
Beneficial	Tomato	*Funneliformis mosseae*	Mycorrhiza	Up	3%	RNAseq	Zouari *et al*. (2014)
Beneficial	California poplar	*Laccaria bicolor*	Mycorrhiza	Variable	0.8%	Microarray	Plett *et al*. (2015)
Beneficial	Grapevine	*Trichoderma harzianum*	Endophyte	Up	1%	Microarray	Perazzolli *et al*. (2012)
Beneficial	*Arabidopsis thaliana*	*Trichoderma harzianum*	Endophyte	Up	<1%	Microarray	Morán‐Diez *et al*. (2012)
Pathogen	Lettuce	*Botrytis cinerea*	Necrotroph	Up	20%	RNAseq	De Cremer *et al*. (2013)
Pathogen	Maize	*Ustilago maydis*	Biotroph	Up	22%	Microarray	Doehlemann *et al*. (2008)
Pathogen	Rice	*Magnaporthe oryzae*	Hemibiotroph	Up	13%	RNAseq	Kawahara *et al*. (2012)

a DEG, the proportion of host differentially expressed genes.

### Endophyte infection alters host primary metabolism

Primary metabolic gene expression was globally downregulated in endophyte‐infected plants relative to uninfected plants, with the exception of cell wall‐associated genes, which tended to be upregulated (Table S2). Many genes involved in transcription were downregulated (Table S4), including those encoding RNA polymerases, TATA‐box binding proteins (TBP) and transcription factors. Consistent with a reduction in expression of genes encoding RNA polymerases, expression of genes involved in RNA processing was downregulated (Table S4). This result suggests that the widespread changes in gene expression may be a consequence of a change in expression of key genes that control transcription, leading to flow‐on effects on expression of over one third of host genes (38%). Many chromatin‐modifying enzymes were downregulated in infected plants, suggesting that changes in chromatin structure may also contribute to the large‐scale changes in expression. Expression of genes involved in nucleotide metabolism was reduced, consistent with a likely decrease in transcriptional activity (Table S5). This reduction in nucleotide metabolism‐related gene expression was confirmed by gene ontology (GO) enrichment analysis, in which the *nucleobase‐containing compound metabolic process* (GO:0006139) term is over‐represented in the set of genes downregulated in infected plants (*P* < 0.0001). Additionally, expression of protein degradation‐related genes was downregulated, consistent with a reduction in expression of genes likely leading to a reduction in protein levels (Table S6).

Given that endophyte‐infected plants generally appear asymptomatic in the absence of stress, it was interesting to find that expression of genes involved in the cell cycle was reduced in infected plants (Table S6). These include cyclins and cyclin‐dependent protein kinases, key regulators of cell cycle progression, and suggests that endophyte infection may slow host growth. This is also supported by a decrease in expression of genes involved in DNA synthesis, chromatin structure and DNA repair (Table S6). Consistent with this observation, infection of perennial ryegrass with *Epichloë festucae* var. *lolii*, previously *Neotyphodium lolii* (Leuchtmann *et al*., 2014), in the absence of environmental stress results in reduced DW (Hahn *et al*., 2008). However, it is likely that endophyte‐induced changes in host cell cycle progression also occur via indirect means. Coincident with endophyte infection, likely leading to reduced host growth, expression of genes encoding for proteins involved in the Krebs cycle (TCA) and glycolysis (Table S7), cell organisation (Table S8), protein targeting and transport processes was downregulated in infected plants (Table S9). Consistent with reduced cell growth, genes encoding for enzymes involved in lipid and carbohydrate metabolism displayed global downregulation, including genes encoding for proteins involved in synthesis of phospholipids, fatty acids, glycerol, and key carbohydrates such as starch, sucrose and galactose (Table S10). Expression of the gene encoding for glycerol‐3‐phosphate dehydrogenase, a key enzyme that links carbohydrate and lipid metabolism, was downregulated. Endophyte infection also had considerable effects on expression of genes encoding proteins involved in cell signalling, with downregulation of genes encoding components of the phosphoinositide, G‐protein and light signalling pathways (Table S11).

Endophyte infection reduced host photosynthesis, with downregulation of genes encoding proteins involved in the Calvin cycle and the tetrapyrrole pathway, which produces chlorophyll (Fig. S1; Table S12). Analysis of the photosynthetic rate of infected and uninfected plants, 14 wk post‐inoculation, confirmed that endophyte infection leads to a significant reduction (*t*_10_ = 2.59, *P* = 0.0271) in photosynthetic rate per leaf DW (Fig. S1), consistent with results seen for *E. festucae* var. *lolii* (Spiering *et al*., 2006). However, comparison of chlorophyll levels in endophyte‐infected and uninfected plants revealed no significant differences (*t*_4_ = 2.00, *P* = 0.116; at 652 nm representative of total chlorophyll content), indicating that the reduction in photosynthetic rate is not due to a reduction in chlorophyll levels.

**Figure 2:**
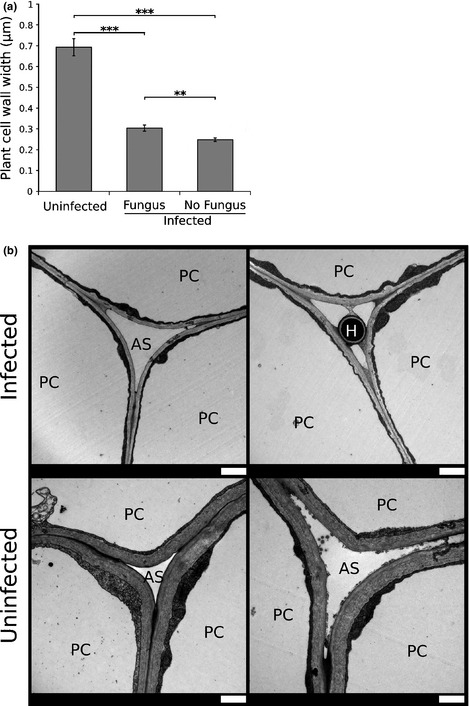
Endophyte infection alters host cell wall thickness. (a) Graph showing differences in plant cell wall width of uninfected perennial ryegrass plants, and endophyte (*Epichloë festucae*) infected plants in regions with and without fungus. Bars represent mean ± SEM. Statistical significance was determined using an unpaired *t*‐test (**, 0.01 ≥ *P* > 0.001; ***, *P* < 0.001). (b) Transmission electron micrographs showing plant cell walls of endophyte‐infected and uninfected plants. H, hypha; PC, plant cell; AS, apoplastic space. Bars, 1 μm.

Contrary to the general downregulation of primary metabolic gene expression, cell wall‐associated genes were upregulated in infected plants. This includes genes encoding proteins involved in synthesis of hemicellulose, cell wall modifiers and cell wall proteins, particularly arabino‐galactan proteins (AGPs) and leucine rich repeat (LRR) proteins (Table S13). This suggests that endophyte infection induces changes in the host cell wall. To test this hypothesis, pseudostem sections from infected and uninfected plants were analysed using transmission electron microscopy (Fig. 2). This analysis revealed that host cell walls in endophyte‐infected plants are dramatically thinner than those from a comparable region in uninfected plants (*t*_121_ = 10.3, *P* = 0.0001 for regions from infected plants with no hyphae; and *t*_124_ = 8.86, *P* = 0.0001 for regions with hyphae). The cell wall of endophyte‐infected plants was thicker in regions adjacent to hyphae than in regions devoid of hyphae (*t*_121_ = 3.24, *P* = 0.0016), implying that the endophyte induces both local and systemic changes in host cell wall structure.

Expression of genes encoding key enzymes involved in biosynthesis and signalling by the phytohormone abscisic acid (ABA) was upregulated in infected plants (Fig. S2; Table S14), suggesting that ABA levels may be elevated in infected plants. These include four putative 9‐*cis*‐epoxycarotenoid dioxygenase (NCED) isoforms, whose genes display dramatic upregulation (from 8‐ to 24‐fold relative to the uninfected control); and a single zeaxanthin epoxidase (ZEP) isoform, whose gene is upregulated two‐fold. The gene for an isoform of abscisic aldehyde oxidase (AAO) was also upregulated 2.4‐fold. However, genes for two additional AAO isoforms were more dramatically downregulated (9‐ and 5‐fold), suggesting that levels of this enzyme may well be reduced. As ABA could not be detected in the apoplastic fluid used for metabolomic analysis, it was not possible to directly determine whether endophyte infection leads to elevated ABA levels. However, as ABA is known to induce stomatal closure (Davies & Zhang, 1991; Trejo *et al*., 1993; Wilkinson & Davies, 2002), stomatal conductance was measured as an indirect indicator of changes in ABA levels (Fig. S2). This revealed that stomatal conductance was significantly reduced in endophyte‐infected plants (*t*_4_ = −6.7, *P* = 0.0025), consistent with the hypothesised increase in ABA levels.

**Figure 3:**
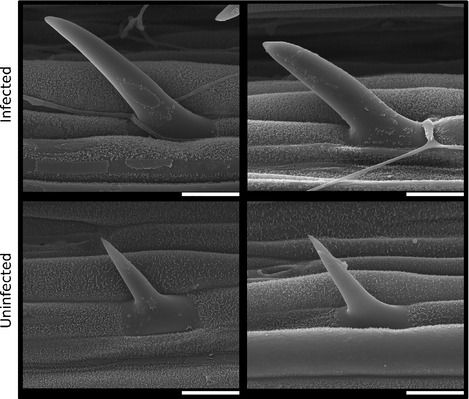
Endophyte infection alters host trichome development. Scanning electron micrographs (independent images) showing differences in trichome size between endophyte (*Epichloë festucae*)‐infected (upper) and uninfected perennial ryegrass plants (lower). Images are from the adaxial leaf surface, *c*. 2 cm from the leaf tip. Bars, 20 μm.

Genes encoding enzymes involved in interconversion of gibberellins between inactive and active forms were upregulated, suggesting that the levels of this group of hormones may also be altered in infected plants (Table S14). As gibberellin promotes the formation of trichomes in *Arabidopsis* (Perazza *et al*., 1998), the effects of endophyte infection on trichome development were examined using scanning electron microscopy (Fig. 3). This revealed a significant increase in the size (area) of trichomes on the adaxial surface of leaves from endophyte‐infected plants (*t*_23_ = 2.90, *P* = 0.0081). There was a trend towards an increase in trichome number on the adaxial leaf surface of endophyte‐infected plants, but this was not statistically significant due to considerable biological variation.

### Endophyte infection increases host secondary metabolic gene expression

The expression of several genes encoding enzymes of the phenylpropanoid biosynthetic pathway, which produces secondary metabolites such as lignin, tannins, flavonoids and anthocyanins, was upregulated in infected plants (Fig. S3; Table S15). This is particularly clear for the early steps in the pathway. For example, genes encoding five putative arogenate dehydratase (ADT) isoforms, three putative isoforms of phenylalanine ammonia lyase (PAL), three putative cinnamic acid 4‐hydroxylase (C4H) isoforms and twelve putative ρ‐coumaroyl‐CoA synthase (4CL) isoforms were all upregulated. Gene expression of four additional key enzymes required for production of lignins, cinnamoyl‐CoA reductase (CCR), cinnamyl alcohol dehydrogenase (CAD), caffeic acid O‐methyltransferase (COMT) and hydroxycinnamoyl‐CoA:skimimate/quinate hydroxycinnamoyltransferase (HCT) was also found to be upregulated. Genes for three out of five differentially expressed putative CCR isoforms, five out of six differentially expressed putative CAD isoforms, a single differentially expressed putative COMT isoform, and 17 out of 18 differentially expressed putative HCT isoforms were upregulated. Genes for three putative isoforms of the enzyme ρ‐coumarate 3‐hydroxylase, which converts coumaroyl shikimic acid to caffeoyl shikimic acid early in the pathway leading to production of guaiacyl and syringyl lignins, were also found to be differentially expressed, with one upregulated and the other two downregulated.

In contrast to the lignin branches, which show mostly upregulation, the anthocyanin branch of the phenylpropanoid pathway displayed more mixed differential expression. Five genes encoding putative chalcone synthase (CHS) enzymes, which catalyse the first step in the anthocyanin branch, were differentially expressed, with four upregulated in infected plants. However, a single chalcone‐flavanone isomerase (CHI) isoform, which catalyses the next step in the pathway, was found to be downregulated in infected plants. Additionally, five genes encoding putative dihydroflavonol 4‐reductase (DFR) isoforms, which catalyse the next step for production of flavonols and the conversion of dihydroflavanols to leucoanthocyanidins were also downregulated. The remaining genes of the anthocyanin branch, leucoanthocyanidin dioxygenase (LDOX), flavanone 3‐hydroxylase (F3H), anthocyanin glycosyltransferase (AGT) and anthocyanin‐specific acyltransferase (ACT) isoforms were all predominantly upregulated.

**Figure 4:**
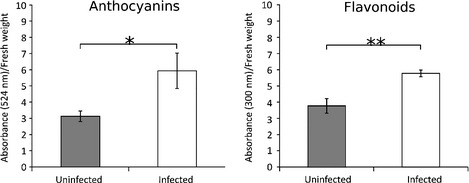
Endophyte infection upregulates phenylpropanoid biosynthesis. Graphs showing differences in anthocyanin and flavonoid levels, as determined by absorbance at 524 nm and 300 nm, respectively, between endophyte (*Epichloë festucae*)‐infected and uninfected perennial ryegrass plants. Bars represent mean ± SEM. Statistical significance was determined using an unpaired *t*‐test (*, 0.05 ≥ *P* > 0.01; **, 0.01 ≥ *P* > 0.001).

Chemical extraction of putative anthocyanins (abs_524nm_) and flavonoids (abs_300nm_) confirmed that there was a significant increase in both the levels of putative anthocyanins (*t*_6_ = 2.46, *P* = 0.0489) and flavonoids (*t*_6_ = 4.04, *P* = 0.00680) in response to endophyte infection (Fig. 4). Genes predicted to be involved in production of proteins for degradation of xenobiotics (foreign chemicals) were upregulated in endophyte‐infected plants (Table S16), suggesting that the host responds to chemicals produced by the fungus.

### Endophyte infection alters host stress responsive gene expression

Infected plants displayed altered expression of stress responsive genes compared to uninfected plants (Tables S17, S18). Abiotic stress response changes were suggestive of perturbed temperature perception, with increased expression of cold‐responsive genes and decreased expression of heat‐responsive genes. Additionally, expression of drought‐related genes was decreased in infected plants. This suggests that rather than enhancing drought tolerance by increasing drought‐related gene expression, endophyte infection instead decreases host sensitivity to drought. This is likely due to increased production of compatible solutes by endophyte‐infected plants, which act as osmoprotectants, limiting host water loss. Expression of genes encoding galactinol synthases, which produce osmoprotective raffinose‐family oligosaccharides, was upregulated in infected plants (Table S10). This is supported by metabolomics analysis of apoplastic fluid, which identified significantly increased levels of the compatible solutes arabitol, threitol, and mannitol in infected plants (Table S19). Consistent with the recent hypothesis that endophytes enhance host drought resistance through increased antioxidant activity (Hamilton & Bauerle, 2012), infected plants displayed increased expression of genes encoding redox‐responsive glutaredoxins and peroxidases (Table S20).

**Figure 5:**
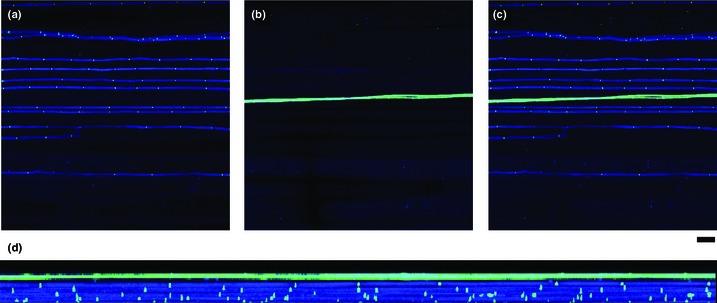
Chitin is masked on hyphae growing inside host tissues. Confocal depth series images of *Epichloë festucae*‐infected perennial ryegrass sheath tissue stained with aniline blue and WGA‐Alexafluor 488. Hyphal cytoplasm stains blue and cell wall appears bright green. (a) Bottom 23 sections of the depth series (7.82 μm) showing hyphae inside the host leaf sheath. (b) Top eight sections of the depth series (2.72 μm) showing hyphae on the surface of the leaf sheath. (c) Merge of the 31 sections of the depth series (10.54 μm). Bar, 20 μm for (a–c). (d) Rotation of the merged depth series to show side‐on view.

Downregulation of genes involved in the response to biotic stress, including putative PR (pathogenesis related) genes, and components of the respiratory burst response (Table S18) was observed in endophyte‐infected plants. This was confirmed by GO analysis, which identified 276 genes with homology to genes possessing a defence‐related GO annotation, of which 34% (*n* = 95) were downregulated in infected plants, compared to 5% (*n* = 15) upregulated. An exception to this downregulation of PR genes was an upregulation of chitinases (*n* = 9). Confocal microscopy analysis reveals that chitin accessible by the lectin wheat germ agglutinin used to stain chitin is present only on epiphyllous (surface) hyphae of *E. festucae*, and at hyphal septa on endophytic hyphae (Fig. 5), suggesting that chitin on intercellular hyphae is somehow masked, protecting it from digestion by plant chitinases.

Consistent with a decreased response to biotic stress, expression of genes encoding proteins involved in biosynthesis and signalling by salicylic acid (SA) was downregulated (Table S14). Genes encoding enzymes involved in synthesis of the defence‐related polysaccharide callose were also downregulated in infected plants, and endo‐1,3‐beta‐glucosidases involved in degradation of callose were upregulated (Table S21). Expression of genes encoding for stress‐responsive WRKY transcription factors was upregulated in infected plants. However, these transcription factors can act as both repressors and activators, so an increase in their expression will have mixed effects on expression of defence‐related genes.

## Discussion

Plants infected with cool‐season grass endophytes, such as *E. festucae*, generally appear asymptomatic in the absence of any biotic or abiotic stress (Schardl, 2001) so it was surprising to find that over 38% of the host genes were differentially expressed between infected and uninfected samples grown under controlled environmental conditions. This is in contrast to a number of other recently analysed beneficial plant–fungal associations in which only *c*. 1–3% of the host gene set is differentially expressed in response to fungal colonisation. These interactions include the mycorrhizal associations between tomato and *Funneliformis mosseae* (Zouari *et al*., 2014), *Lotus japonicus* and *Gigaspora margarita* (Guether *et al*., 2009), *Medicago truncatula* and *Glomus intraradices* (Gomez *et al*., 2009), rice and *Glomus intraradices* (Güimil *et al*., 2005), and *Trichoderma* interactions with grapevine (Perazzolli *et al*., 2012) and *Arabidopsis* (Morán‐Diez *et al*., 2012). In the association between rice and *G. intraradices*, only *c*. 0.5% of the analysed host genes displayed altered expression upon infection (Güimil *et al*., 2005). Interestingly, one of the genes upregulated in this association encodes a WRKY transcription factor, which is also upregulated when tomato is colonised by the phytopathogens *Magnaporthe oryzae* and *Fusarium moniliforme*, and is speculated to be a transcription factor involved in the basic plant defence response to many different fungal infections (Güimil *et al*., 2005). This view is supported by our study, in which the homologous ryegrass *WRKY* gene (m.60140) is also upregulated in response to endophyte infection. In contrast to beneficial associations, plant–pathogenic associations tend to induce more significant changes in host gene expression, similar to that seen for *Epichloë*. For example, in the interactions between lettuce and *Botrytis cinerea* (De Cremer *et al*., 2013), and maize and *Ustilago maydis* (Doehlemann *et al*., 2008), *c*. 20% of the host genes are differentially expressed; and in the rice–*Magnaporthe oryzae* interaction over 10% of host genes are altered (Kawahara *et al*., 2012).

In contrast to the aforementioned mycorrhizal interactions, where almost all of the differentially expressed host genes are upregulated in response to fungal infection, the majority of the differentially expressed ryegrass genes were downregulated. Many of these genes encode proteins involved in primary metabolism, implying that endophyte infection represses primary metabolism. This is consistent with the results of Ambrose & Belanger (2012), which showed a general downregulation of genes involved in primary metabolism in response to infection of red fescue by *E. festucae*. In support of the hypothesis that endophyte infection represses primary metabolism, photosynthesis is reduced in infected plants. Surprisingly, this reduction in photosynthesis is contrary to the results of Ambrose & Belanger (2012), where genes involved in photosynthesis were found to be upregulated in endophyte‐infected red fescue. However, a reduction in photosynthesis is consistent with the results of Spiering *et al*. (2006), which showed that infection of *L. perenne* by *E. festucae* var. *lolii* leads to reduction in photosynthetic activity. This suggests that the effects of endophyte infection on host photosynthesis may vary depending on the endophyte and/or host species.

This result suggests that the plant is under biotic stress (Bilgin *et al*., 2010). In contrast to the reduction of photosynthesis, expression of secondary metabolism genes, particularly those encoding for enzymes producing bioprotective phenylpropanoids, is elevated. This observation suggests that endophyte‐infection leads to a reprogramming of host metabolism, or redistribution of resources, towards secondary metabolism, at a cost to primary metabolism. Recent work in the *U. maydis*–maize association has revealed that a fungal chorismate mutase enters host cells, leading to a reprogramming of host metabolism (Djamei *et al*., 2011). However, *E. festucae* does not appear to possess a homologue of this secreted chorismate mutase, suggesting that it reprograms the host via an alternative method. Generally, the alternative branches of the phenylpropanoid biosynthesis pathway (lignin and anthocyanin) display different regulation states depending on the stress sensed by the plant. For example, wounding induces upregulation of the genes involved in the lignin biosynthesis, whereas low temperatures induce higher production of anthocyanins (Dixon & Paiva, 1995). Here, a general upregulation of both branches of the phenylpropanoid pathway is observed. This could be due to the fact that different tissue types were included in the samples used for RNAseq, including tissues both in contact with and distant from fungal hyphae.

Contrary to the general downregulation of primary metabolism, genes associated with cell wall synthesis were upregulated in response to fungal infection. However, cell wall thickness was reduced in endophyte‐infected plants relative to uninfected plants. This may be due to enzymatic digestion of the host cell wall by the fungus, leading to reduced cell wall thickness despite the fact that expression of wall‐associated genes is upregulated. In support of this hypothesis, the genes encoding for a pectin methylesterase (PME) and endoxylanase are very highly expressed by *E. festucae in planta* (Eaton *et al*., 2010, 2015). The presence of a thicker cell wall adjacent to hyphae in infected plants suggests that some of the changes in wall‐associated gene expression may be required for physical attachment of hyphae to the host cell wall (Christensen *et al*., 2002). The fungal PME may play a role in this, as demethylesterification of pectin by the PME can facilitate crosslinking of fungal and host cell walls (Pelloux *et al*., 2007). Interestingly, in the synthetic obligate mutualistic association between the alga *Chlamydomonas reinhardtii* and the fungus *Aspergillus nidulans*, algal cell walls in contact with fungal hyphae are thinner, possibly due to the activity of fungal secreted cell wall remodelling enzymes (Hom & Murray, 2014). As endophyte infection seems to slow host growth (Hahn *et al*., 2008), the cell wall thickness differences may also be due to different developmental stages in infected vs uninfected plants. In order to minimise the influence of this factor, comparable sections were harvested for each plant. Moreover, the measured plants were mature (11 wk post‐inoculation), and at this stage development differences tend to be minimal. Future studies will investigate whether this effect is also seen in seed infected (naturally infected) plants or other *Epichloë*–grass associations to determine whether this result is a feature of this particular association or a more general consequence of the symbiosis between *Epichloë* endophytes and their hosts.

Despite major differences between endophyte and mycorrhizal effects on host gene expression, our study has identified a key similarity with respect to upregulation of genes encoding enzymes involved in biosynthesis of the phytohormone gibberellin in response to infection by both groups of fungi (Gomez *et al*., 2009). Mycorrhizal colonisation has been shown to be enhanced in gibberellin‐deficient mutant hosts (Foo *et al*., 2013), suggesting that host gibberellin restricts mycorrhizal growth *in planta*, the opposite of the role that plant strigolactones play in inducing branching of mycorrhizal fungi (Akiyama & Hayashi, 2006). Given that we have shown here that gibberellin biosynthetic genes are upregulated in response to endophyte infection, it is possible that gibberellin plays a role in controlling endophyte growth *in planta*. This putative upregulation of gibberellin signalling in endophyte‐infected plants is supported by the dramatic effect of infection on trichome development, as trichome development is controlled by gibberellin signalling in *Arabidopsis* (Perazza *et al*., 1998).

In addition to changes in hormone signalling, endophyte infection dramatically altered host pathways involved in the response to abiotic and biotic stresses. Drought‐related gene expression was lower in infected plants. However, levels of compatible solutes were increased, suggesting that endophyte infection may enhance drought tolerance by priming the host with compatible solutes to avoid imposition of drought stress. Additionally, larger trichomes and increased stomatal closure in endophyte‐infected plants will likely increase moisture retention.

The increased production of compatible solutes is also linked to the altered temperature perception observed in infected plants, as genes encoding enzymes involved in production of raffinose family oligosaccharides, which act as compatible solutes and accumulate during cold acclimation in rye (Koster & Lynch, 1992), are upregulated in infected plants. Endophyte infection does not induce any obvious host defence response. Thus, it was hypothesised that either the fungal pathogen associated molecular patterns (PAMPs) are masked such that the fungus is not detected by the host, or that host defence is downregulated by the fungus or by the host itself (Schardl *et al*., 2004). Our study revealed downregulation of many genes involved in the host biotic stress response in endophyte‐infected plants, supporting the latter two hypotheses. This is in contrast to *E. festucae* symbiotic mutants that have a pathogen‐like interaction with the host, and a dramatic induction of host defence‐related gene expression (Eaton *et al*., 2010, 2015). Small secreted proteins (SSPs) produced by the fungus are predicted to play a role in downregulating host defence and controlling mutualism, similar to the recently identified SSP of *Laccaria bicolor*, which is essential for establishment of a mutualistic interaction with poplar (Plett *et al*., 2011). Interestingly, many of the most highly expressed *E. festucae* genes *in planta* encode putative SSPs (Eaton *et al*., 2010, 2015).

In an exception to the downregulation of defence‐related genes, plant chitinase expression was elevated in endophyte‐infected plants. Moreover, one of the most highly expressed *E. festucae* genes *in planta* encodes a chitinase (Eaton *et al*., 2010, 2015). Chitin oligomers released via the action of chitinases are potent elicitors of host defence (Kaku *et al*., 2006). However, *E. festucae* cell wall chitin appears to be masked when this fungus grows *in planta*, a result similar to the phytopathogen *Cladosporium fulvum* (van Esse *et al*., 2007; de Jonge *et al*., 2010). The highly expressed *E. festucae* SSPs may play a role in this masking, as they do in *C. fulvum*. Interestingly, chitinases are also upregulated in mycorrhizal associations (Gomez *et al*., 2009). Given the apparent downregulation of host defence‐related gene expression, endophyte infection would be expected to increase susceptibility to microbial pathogens. However, this is not the case as infection by *E. festucae* var. *lolii*, has been shown to increase resistance to fungal pathogens (Tian *et al*., 2008; Pańka *et al*., 2013a) due to production of phenolic compounds (Pańka *et al*., 2013a). This is consistent with the observed increased expression of several genes encoding enzymes of the phenylpropanoid pathway, which produces these bioprotective phenolics in infected plants. This suggests that whereas *E. festucae* infection leads to downregulation of genes directly involved in host defence, such as PR genes, there is likely an upregulation of the phenylpropanoid pathway and chitinases that would provide enhanced resistance to other microbes, possibly as a means of competitive exclusion. In the interaction between the biotrophic fungus *U. maydis* and maize, a fungal effector protein has been shown to enter host cells and activate genes encoding enzymes of the phenylpropanoid pathway, leading to an increase in anthocyanins and a decrease in lignin (Tanaka *et al*., 2014). This is hypothesised to increase virulence by redistributing phenylpropanoid intermediates away from the production of host defence‐related compounds (phytoalexins) towards anthocyanin production. Despite being a mutualist, it is possible that *E. festucae* similarly upregulates host anthocyanin and flavonoid levels in order to reduce host production of defence compounds.

This study uncovers the dramatic effects of endophyte infection on ryegrass gene expression. Endophyte infection has important effects not only on host responses to stress, but also leads to reprogramming of host metabolism, and substantially alters host development. Understanding the molecular basis for these endophyte‐induced plant changes will be a major focus of future research.

## Supporting information

Please note: Wiley Blackwell are not responsible for the content or functionality of any supporting information supplied by the authors. Any queries (other than missing material) should be directed to the *New Phytologist* Central Office.

**Fig. S1** Effects of endophyte infection on the tetrapyrrole biosynthetic pathway.
**Fig. S2 ** Endophyte infection upregulates the abscisic acid biosynthetic pathway.
**Fig. S3** Endophyte infection upregulates the phenylpropanoid biosynthetic pathway.

**Table S1** Results of the automatic annotation of the ryegrass ORFs

**Table S2** Number of upregulated and downregulated genes for all pathways described in Mercator

**Table S3** Comparison of Nanostring and RNAseq ratios

**Table S4** Annotations of the genes predicted to encode for enzymes involved in RNA metabolism (RNA transcription, regulation of transcription, RNA processing)

**Table S5** Annotations of the genes predicted to encode for enzymes involved in nucleotide metabolism

**Table S6** Annotations of the genes predicted to encode for enzymes involved in protein degradation, cell cycle, DNA synthesis and DNA repair

**Table S7** Annotations of the genes predicted to encode for enzymes involved in the TCA cycle and glycolysis

**Table S8** Annotations of the genes predicted to encode for enzymes involved in cell organisation

**Table S9** Annotations of the genes predicted to encode for enzymes involved in protein targeting, transport and vesicle transport

**Table S10** Annotations of the genes predicted to encode for enzymes involved in major carbohydrate metabolism and lipid metabolism

**Table S11** Annotations of the genes predicted to encode for enzymes involved in signalling (light, G‐protein and phosphoinositide)

**Table S12** Annotations of the genes predicted to encode for enzymes involved in tetrapyrrole synthesis and photosynthesis

**Table S13** Annotations of the genes predicted to encode for enzymes involved in cell wall metabolism

**Table S14** Annotations of the genes predicted to encode for enzymes involved in hormone metabolism

**Table S15** Annotations of the genes predicted to encode for enzymes involved in secondary metabolism

**Table S16** Annotations of the genes predicted to encode for enzymes involved in degradation of xenobiotics

**Table S17** Annotations of the genes predicted to encode for enzymes involved in abiotic stress responses

**Table S18** Annotations of the genes predicted to encode for enzymes involved in biotic stress responses

**Table S19** Results of the metabolomic analysis of the apoplastic fluid by GC‐MS

**Table S20** Annotations of the genes predicted to encode for enzymes involved in redox reactions

**Table S21** Annotations of the genes predicted to encode for enzymes involved in callose and endo‐1,3‐beta glucosidase synthesis
