# Supplementary material for: Fungal endophyte infection of ryegrass reprograms host metabolism and alters development
Source: New Phytol. 2015 Aug 25;208(4):1227–40. doi: 10.1111/nph.13614 (PMC5049663; doi:10.1111/nph.13614)
Supplement: Supplementary file 1 — Fig. S1 Effects of endophyte infection on the tetrapyrrole biosynthetic pathway. Fig. S2 Endophyte infection upregulates the abscisic acid biosynthetic pathway. Fig. S3 Endophyte infection upregulates the phenylpropanoid biosynthetic pathway. [file NPH-208-1227-s001.pdf]

New Phytologist

***New Phytologist* Supporting Information**

Article title: **Fungal endophyte infection of ryegrass reprograms host metabolism and alters development**

Authors: Pierre-Yves Dupont, Carla J. Eaton, Jason J. Wargent, Susanne Fechtner, Peter Solomon, Jan Schmid, Robert C. Day, Barry Scott and Murray P. Cox

Article acceptance date: 18 July 2015

The following Supporting Information is available for this article:

Fig. S1 Effects of endophyte infection on the tetrapyrrole biosynthetic pathway

A. Schematic representation of the tetrapyrrole biosynthetic pathway (Moulin & Smith, 2005), with red text/arrows indicating the positions of down-regulated genes. GlnRS, glutaminyl-tRNA synthetase; PBGD, porphobilinogen deaminase; UROS, uroporphyrinogen III synthase; UROD, uroporphyrinogen III decarboxylase; CPOX, coproporphyrinogen oxidase; PPOX, protoporphyrinogen oxidase; MgCh, magnesium chelatase; FeCh, ferrochelatase; HO, haem oxygenase; PΦS, phytochromobilin synthase; SirB, sirohydrochlorin ferrochelatase. **B**. Graph showing difference in photosynthetic rate per dry weight (μmol CO_2 m^{-2} s^{-1}) between endophyte-infected and uninfected plants. Bars represent mean ± SEM. Statistical significance was determined using an unpaired *t*-test (*, $0.05 \geq P > 0.01$).

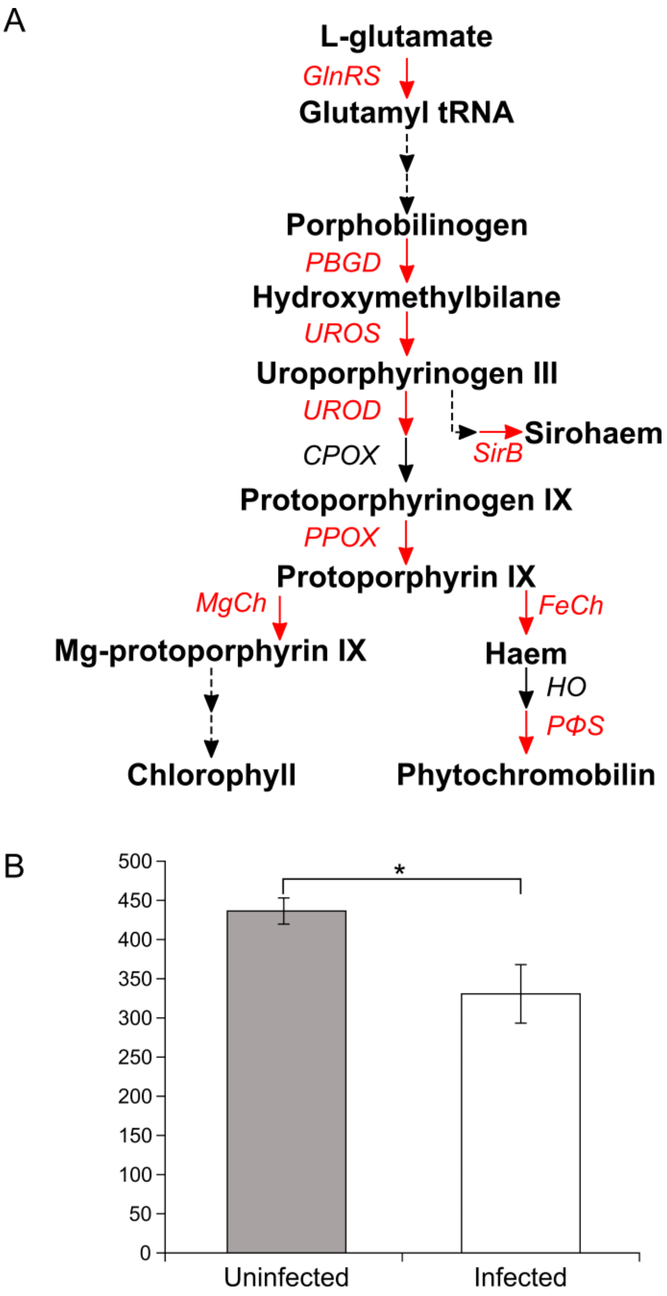
A
L-glutamate
GlnRS
Glutamyl tRNA
Porphobilinogen
PBGD
Hydroxymethylbilane
UROS
Uroporphyrinogen III
UROD
Sirohaem
SirB
CPOX
Protoporphyrinogen IX
PPOX
Protoporphyrin IX
MgCh
FeCh
Mg-protoporphyrin IX
Haem
HO
PΦS
Chlorophyll
Phytochromobilin
B
500
450
400
350
300
250
200
150
100
50
0
*
Uninfected
Infected

Fig. S2 Endophyte infection up-regulates the abscisic acid biosynthetic pathway

(a) Pathway schematic showing changes in expression of key genes in the abscisic acid biosynthetic pathway (Schwartz *et al.*, 2003). Coloured arrows are indicative of gene expression changes. Green arrow, up-regulated in infected plants; red arrow, down-regulated in infected plants. ZEP, zeaxanthin epoxidase; VDE, violaxanthin de-epoxidase; NSY, neoxanthin synthase; NCED, 9-cis-epoxycarotenoid dioxygenase; ABA2, abscisic acid 2; AAO, abscisic aldehyde oxidase; MoCo, molybdenum cofactor. **(b)** Graph showing difference in stomatal conductance (g/fresh weight) between endophyte-infected and uninfected plants. Bars represent mean ± SEM. Statistical significance was determined using an unpaired *t*-test (**, 0.01≥*P*>0.001).

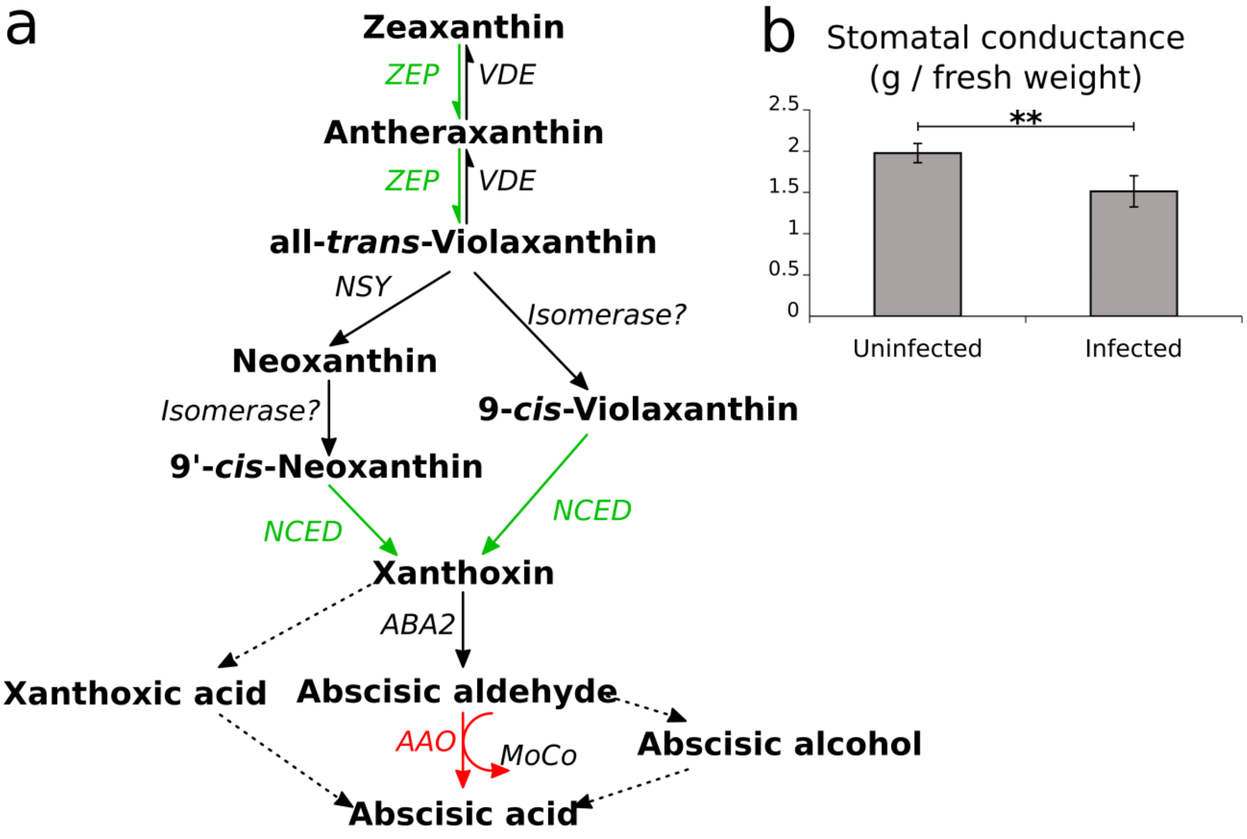

Fig. S3 Endophyte infection up-regulates the phenylpropanoid biosynthetic pathway

Pathway schematic showing changes in expression of key genes in the phenylpropanoid biosynthetic pathway (Peng *et al.*, 2008). Coloured arrows are indicative of gene expression changes. Green arrow, up-regulated in infected plants; red arrow, down-regulated in infected plants. CM, chorismate mutase; PAT, prephenate amino transferase; ADT, arogenate dehydratase; ADH, arogenate dehydrogenase; Trp, tryptophan; Tyr, tyrosine; Phe, phenylalanine; PAL, phenylalanine ammonia lyase; C4H, cinnamic acid 4-hydroxylase; 4CL, ρ-coumaroyl-CoA synthase; CHS, chalcone synthase; CHI, chalcone-flavanone isomerase; F3H, flavanone 3-hydroxylase; DFR, dihydroflavonol 4-reductase; LDOX, leucoanthocyanidin dioxygenase; AGT, anthocyanin glycosyltransferase; ACT, acyltransferase; FLS, flavonol synthase; HCT, hydroxycinnamoyl-CoA:skimimate/quinate hydroxycinnamoyltransferase; CCR, cinnamoyl-CoA reductase; CAD, cinnamyl alcohol dehydrogenase; C3H, ρ-coumarate 3-hydroxylase; CCoAOMT, caffeoyl-CoA 3-O-methyltransferase; COMT, caffeic acid O-methyltransferase.

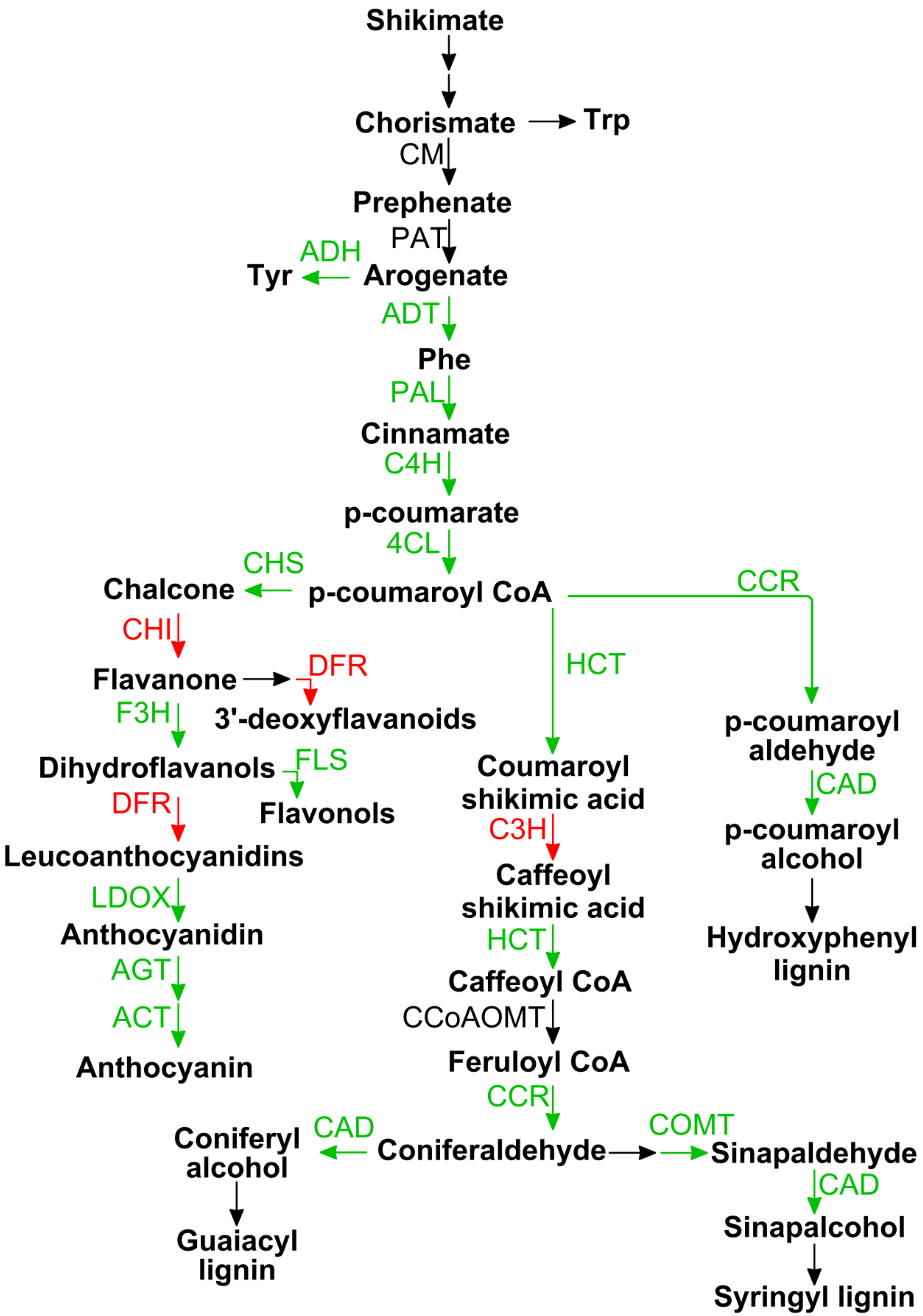
Shikimate
Chorismate
Trp
CM
Prephenate
PAT
ADH
Tyr
Arogenate
ADT
Phe
PAL
Cinnamate
C4H
p-coumarate
4CL
CHS
Chalcone
p-coumaroyl CoA
CCR
CHI
Flavanone
DFR
HCT
3'-deoxyflavanoids
F3H
p-coumaroyl aldehyde
Dihydroflavanols
FLS
Coumaroyl shikimic acid
CAD
DFR
Flavonols
p-coumaroyl alcohol
C3H
Leucoanthocyanidins
Caffeoyl shikimic acid
LDOX
Hydroxyphenyl lignin
Anthocyanidin
HCT
AGT
Caffeoyl CoA
ACT
CCoAOMT
Anthocyanin
Feruloyl CoA
CCR
CAD
COMT
Coniferyl alcohol
Coniferaldehyde
Sinapaldehyde
CAD
Sinapalcohol
Guaiacyl lignin
Syringyl lignin

References

Moulin M, Smith A. **2005**. Regulation of tetrapyrrole biosynthesis in higher plants. *Biochemical Society Transactions* **33**: 4–7.

Peng M, Hudson D, Schofield A, Tsao R, Yang R, Gu H, Bi Y-M, Rothstein SJ. **2008**. Adaptation of *Arabidopsis* to nitrogen limitation involves induction of anthocyanin synthesis which is controlled by the NLA gene. *Journal of Experimental Botany* **59**: 2933–2944.

Schwartz S, Qin X, Zeevaart J. **2003**. Elucidation of the indirect pathway of abscisic acid biosynthesis by mutants, genes, and enzymes. *Plant Physiology* **131**: 1591–1601.

Tables S1–S21 – see separate Excel files

Table S1 Results of the automatic annotation of the ryegrass ORFs

Table S2 Number of up-regulated and down-regulated genes for all pathways described in Mercator

Table S3 Comparison of Nanostring and RNAseq ratios

Table S4 Annotations of the genes predicted to encode for enzymes involved in RNA metabolism (RNA transcription, regulation of transcription, RNA processing)

Table S5 Annotations of the genes predicted to encode for enzymes involved in nucleotide metabolism

Table S6 Annotations of the genes predicted to encode for enzymes involved in protein dgradation, cell cycle, DNA synthesis and DNA repair

Table S7 Annotations of the genes predicted to encode for enzymes involved in the TCA cycle and glycolysis

Table S8 Annotations of the genes predicted to encode for enzymes involved in cell organisation

Table S9 Annotations of the genes predicted to encode for enzymes involved in protein targeting, transport and vesicle transport

Table S10 Annotations of the genes predicted to encode for enzymes involved in major carbohydrate metabolism and lipid metabolism

Table S11 Annotations of the genes predicted to encode for enzymes involved in signalling (light, G-protein and phosphoinositide)

Table S12 Annotations of the genes predicted to encode for enzymes involved in tetrapyrrole synthesis and photosynthesis

Table S13 Annotations of the genes predicted to encode for enzymes involved in cell wall metabolism

Table S14 Annotations of the genes predicted to encode for enzymes involved in hormone metabolism

Table S15 Annotations of the genes predicted to encode for enzymes involved in secondary metabolism

Table S16 Annotations of the genes predicted to encode for enzymes involved in degradation of xenobiotics

Table S17 Annotations of the genes predicted to encode for enzymes involved in abiotic stress responses

Table S18 Annotations of the genes predicted to encode for enzymes involved in biotic stress

responses

Table S19 Results of the metabolomic analysis of the apoplastic fluid by GC-MS

Table S20 Annotations of the genes predicted to encode for enzymes involved in redox reactions

Table S21 Annotations of the genes predicted to encode for enzymes involved in callose and endo-1,3-beta glucosidase synthesis
